# Supplementary material for: Highly efficient octave-spanning long-wavelength infrared generation with a 74% quantum efficiency in a χ(2) waveguide
Source: Nat Commun. 2023 Nov 6;14:7125. doi: 10.1038/s41467-023-42912-0 (PMC10628208; doi:10.1038/s41467-023-42912-0)
Supplement: Supplementary file 1 — Supplementary Information [file 41467_2023_42912_MOESM1_ESM.pdf]

# Highly efficient octave-spanning long-wavelength infrared generation with a 74% quantum efficiency in a $\chi^{(2)}$ waveguide

**Bo Hu[1, 6], Xuemei Yang[1, 6], Jiangen Wu[2, 6], Siyi Lu[1], Hang Yang[1], Zhe Long[1], Linzhen He[1], Xing Luo[3], Kan Tian[1], Weizhe Wang[1], Yang Li[1], Han Wu[1]*, Wenlong Li[4], Chunyu Guo[3], Huan Yang[2]*, Qi Jie Wang[5] and Houkun Liang[1]***

*[1] School of Electronics and Information Engineering, Sichuan University, Chengdu, Sichuan 610064, China*

*[2] Sino-German College of Intelligent Manufacturing, Shenzhen Technology University, Shenzhen, Guangdong 518118, China*

*[3] College of Physics and Optoelectronic Engineering, Shenzhen University, Shenzhen 518060, China*

*[4] Chengdu Dien PHOTOELECTRIC Technology Co., Ltd. Chengdu, Sichuan 610100, China*

*[5] School of Electrical & Electronic Engineering & The Photonics Institute, Nanyang Technological University 639798, Singapore, Singapore*

*[6] These authors contributed equally: Bo Hu, Xuemei Yang, Jiangen Wu*

## I. Supplementary Note 1: The choice and generalization of nonlinear crystals used for the birefringence $\chi^{(2)}$ waveguide

Nonlinear crystals including oxide and non-oxide crystals are generally used in the mid-infrared parametric conversion. Particularly, non-oxide crystals are usually employed for long-wavelength infrared (LWIR) generation. Representatively, $ZnGeP_2$ [1], $AgGaS_2$ [2], GaSe [3], and $CdSiP_2$ [4] crystals have been extensively studied. Here, the choice and generalization of nonlinear crystals used for the birefringence $\chi^{(2)}$ waveguide are discussed.

In principle, $AgGaS_2$, GaSe, and $CdSiP_2$ and other nonlinear crystals could also be effectively processed by the ultrafast laser direct writing (ULDW) technique as birefringence $\chi^{(2)}$ waveguide platforms [5]. Here, ZGP is used with reasons summarized as following aspects. Firstly, ZGP has an excellent quadratic nonlinear coefficient ($d_{36}$ ~ 75 pm/V) which is higher than those of $AgGaS_2$ and GaSe, only lower than that of $CdSiP_2$ ($d_{36}$ ~ 84.5 pm/V); however, the transparent window of ZGP is broader than that of $CdSiP_2$ (0.74~12 μm for ZGP v.s. 0.5~9 μm for $CdSiP_2$). Secondly, high quality ZGP crystals cut into designed phase-matching (PM) angles are easy to be obtained with mature growth technologies. On the contrary, GaSe with a broader transparent window could be cleaved only along the (001) plane (z-cut, $\theta = 0^{\circ}$), which restricts its usefulness in birefringence $\chi^{(2)}$ waveguide. In addition, ZGP has a good thermal conductivity (35 W/mK) which eliminates possible thermal related variations of the fabricated $\chi^{(2)}$ waveguide, in case there is detrimental absorption. (In principle there is no obvious absorption loss from the ZGP waveguide.). With these considerations, we choose ZGP as a typical example of birefringence $\chi^{(2)}$ waveguide for highly efficient and broadband LWIR light generation.

Besides $AgGaS_2$, GaSe, and $CdSiP_2$, nonlinear non-oxide crystals with large bandgap energy, such as $LiGaS_2$ [6, 7] may also be fabricated into a $\chi^{(2)}$ waveguide through the ULDW technique, which could be pumped at ~ 1 μm wavelength. This would broaden the usefulness and impact of demonstrated birefringence $\chi^{(2)}$ waveguide. **Supplementary Table 1** summarizes and compares the optical properties of some typical long-wavelength IR nonlinear crystals.

**Supplementary Table 1** Summary and comparison of the optical properties of some typical long-wavelength infrared nonlinear crystals.

| Crystals | Point group | Nonlinear coefficient (pm/V) | Transparent range (μm) | Thermal conductivity (W/mK) | Bandgap (ev) | Reference |
|---|---|---|---|---|---|---|
| ZGP | 42m | 75 | 0.74-12 | 35 | 2.1 | 1 |
| $AgGaS_2$ | 42m | 12 | 0.47-13 | 1.4 | 2.7 | 2 |
| GaSe | 62m | 57 | 0.65-18 | 2.0 | 2.1 | 3 |
| $CdSiP_2$ | 42m | 84.5 | 0.5-9 | 13.6 | 2.45 | 4 |
| $LiGaS_2$ | mm2 | 5.8 | 0.32-11.6 | 5.1 | 4.15 | 6, 7 |

## II. Supplementary Note 2: Characterization of the signal wave

Here, the corresponding signal information related to idler is presented. As shown in the Supplementary Figure 1(a), the relevant signal spectrum is measured and plotted together with the idler spectrum. The signal spectrum has a central wavelength located at ~ 3.45 μm, as predicted by the PM condition. The output power of the signal wave as a function of the pump power is measured and displayed in Supplementary Figure 1(b). The maximum unsaturated signal power of 1.6 mW is obtained at a pump power of 3.2 mW, indicating a power conversion efficiency of ~ 50% and a quantum efficiency of 72% which is very similar to that of the idler efficiency measurement. Meanwhile, in the low gain region, the signal power is comparable with that of idler power, revealing an inherent characteristic of OPG seeded by quantum noises [8].

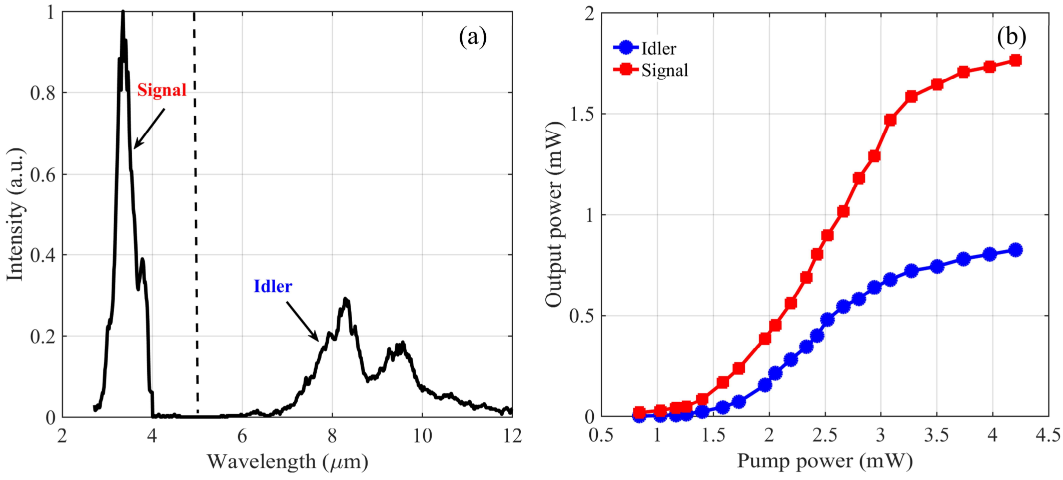


**Supplementary Figure 1.** The measured signal and idler spectra and power from the 10-mm-long ZGP $\chi^{(2)}$ birefringence waveguide. (a) The spectra of signal and idler waves. (b) The output power of signal and idler waves as a function of pump power.

## III. Supplementary Note 3: the OPG coherence measurement and $\chi^{(3)}$ nonlinearity effect characterization

In this section, a qualitative analysis of $\chi^{(3)}$ effect on OPG process and OPG output coherence when light propagating through the ZGP waveguide is investigated.

To investigate the $\chi^{(3)}$ effect such as self-phase modulation (SPM) and four-wave mixing (FWM) in the birefringence $\chi^{(2)}$ waveguide, the comparison of measured incident and transmitted pump spectrum is presented, as shown in Supplementary Figure 2(a). No obvious spectral broadening or sideband generation is observed when a 2.4 pump pulse with a pulse energy 6 nJ and a pulse duration of 320 fs is focused by a $CaF_2$ lens with a focal length of 40 mm into the ZGP waveguide. It indicates that $\chi^{(3)}$ effects such as SPM or FWM are negligible as the pump pulse propagating along the $\chi^{(2)}$ ZGP waveguide. In addition, a simulation of OPG in the $\chi^{(2)}$ ZGP waveguide is conducted based on coupled-wave equations with random noises as the input signal [9, 10]. The simulated idler spectra with and without including the $\chi^{(3)}$ nonlinearity are compared, as shown in Supplementary Figure 2(b). Nearly identical spectra are obtained which means in the $\chi^{(2)}$ ZGP waveguide, $\chi^{(3)}$ effects do not make significant contribution to the parametric process.

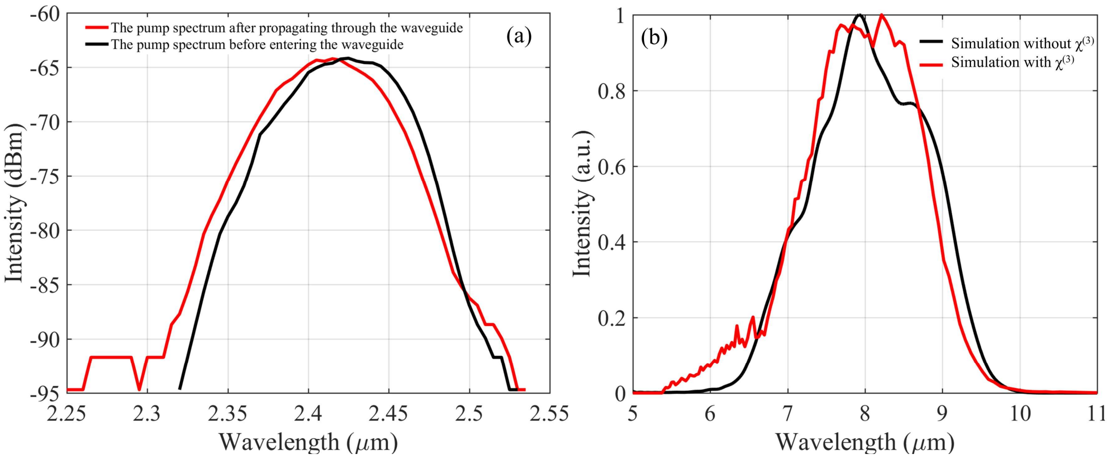


**Supplementary Figure 2.** Characterization of $\chi^{(3)}$ effect in the $\chi^{(2)}$ ZGP waveguide. (a) Comparison of the measured pump spectra before entering and after propagating through the $\chi^{(2)}$ ZGP waveguide. (b) Comparison of the simulated idler spectra with and without $\chi^{(3)}$ effects.

Limited by the MIR output power from the birefringence $\chi^{(2)}$ waveguide, it is difficult to directly measure the OPG coherence. Alternatively, we reproduce the OPG experiment in a bulk ZGP crystal (DPT, YS-ZGP cut at $\theta$ =48.4º, $\varphi$ =0º) with the same experimental condition for a qualitatively measurement. Similar idler spectra are produced from the bulk ZGP crystal and the ZGP $\chi^{(2)}$ waveguide.

A home-built second-harmonic generation-based interferometer autocorrelator (IAC) is used to measure the temporal profile of the generated idler pulse. As presented in Supplementary Figure 2(a), an interferometric trace with a 1:8 ratio between the background and the maximum of the IAC signal proves the reliability of the measurement result. The electric field is reconstructed through a genetic algorithm based on the "evolutionary phase retrieval from interferometric autocorrelation (EPRIAC)" algorithm [11]. In the Taylor expansion of the reconstructed spectral phase, dispersion terms up to seventh order are considered. The retrieved IAC trace exhibiting a good match with the measured one and the reconstructed temporal profiles are shown in Supplementary Figure 3(a, b), respectively. A pulse width of ~ 270 fs is measured, which is much longer than the transform-limited pulse width (~ 60 fs) and similar to the duration of the pump pulse (320 fs). Moreover, pulse splitting in some extent is revealed. The measurement thus verifies that the output of OPG is partially coherent, which is consistent with the previous reported works [12-14]. Meanwhile, considering the negligible $\chi^{(3)}$ effect on the formation of idler wave from the designed waveguide, as demonstrated in Supplementary Figure 2, it is suggested that in the $\chi^{(2)}$ ZGP waveguide with only 10 mm in length and multi-wavelength-scale waveguide cross-section dimensions, the OPG output has the similar coherence and pulsing characteristics, as measured in Supplementary Figure 3.

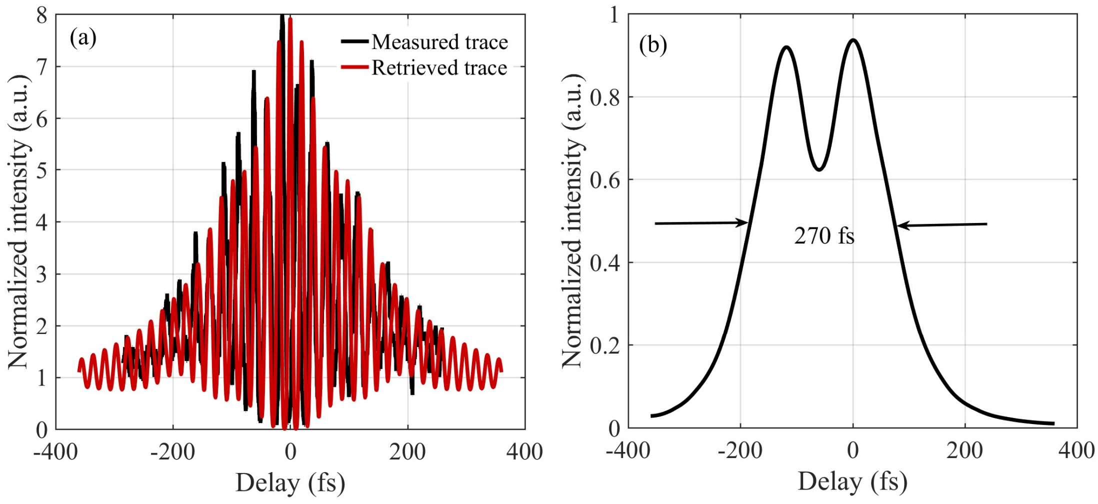


**Supplementary Figure 3.** The temporal characterization by reproducing the OPG experiment in a 10-mm-long bulk ZGP crystal with the same experimental condition of the $\chi^{(2)}$ ZGP waveguide. (a) The measured (black) and retrieved (red) IAC traces. (b) The retrieved temporal profile shows 270 fs pulse width and certain pulse splitting.

## IV. Supplementary Note 4: Proof-of-concept study of c. w. pumped DFG in $\chi^{(2)}$ ZGP waveguide

As the feasibility study of c. w. pumped $\chi^{(2)}$ frequency down-conversion in the birefringence ZGP $\chi^{(2)}$ waveguide, benefited from the employed ZGP crystal (manufactured by DIEN TECH, YS-ZGP) which exhibits much lower transmission loss in the near-infrared region compared to that of traditional ZGP crystal as shown in Supplementary Figure 4(a) and (b), a tunable c. w. DFG across the mid-infrared band in a new ZGP waveguide driven by near-infrared fiber lasers is feasible and demonstrated.

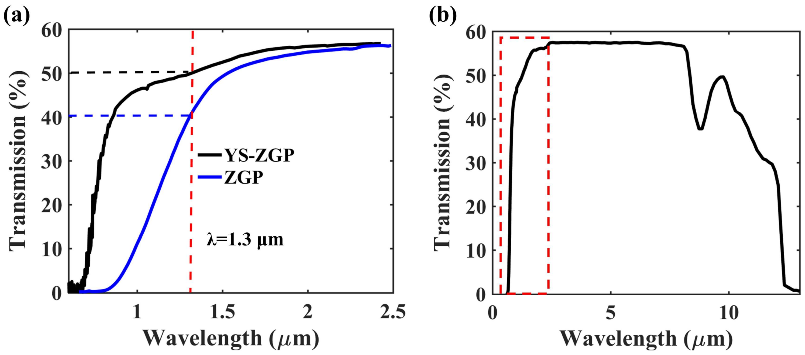


**Supplementary Figure 4. (a)** The comparison of measured transmission spectrum between 8-mm-thick YS-ZGP crystal and traditional ZGP crystal with the same thickness across the wavelength range of 0.5 - 2.5 μm. (b) The measured transmission spectrum of the 8-mm-thick YS-ZGP crystal across the wavelength range of 0.5 - 12 μm (The Fresnell reflection is not subtracted). The enlargement of the marked red frame is plot in Fig. 4(a).

The experimental setup of c. w. LWIR DFG in ZGP waveguide is mainly composed of three parts, including a home-built c. w. tunable random Raman fiber laser (RRFL) which is used as DFG pump source, as depicted in Supplementary Figure 5, a commercial erbium-doped fiber amplifier (EDFA) seeded by tunable signal-frequency DFB laser (Conquer, KG-TLS-13-P-FA) emitting at the spectral range of 1527-1567 nm as DFG signal source and a newly fabricated ZGP waveguide. The RRFL and EDFA source are combined by a fiber wavelength division multiplexer (WDM) to form an all-fiber laser source for driving DFG process in the ZGP waveguide. Detailed configuration of RRFL is presented at Supplementary Figure 5. A wavelength tunable filter with 0.1 nm -3 dB bandwidth is integrated into a 1:1 coupler (I)-based fiber loop mirror to provide the wavelength-selectable point feedback. Combining with the wavelength-selectable point feedback and the random distributed Rayleigh feedback in 5 km-long single mode fiber, 1040-1090 nm tunable ytterbium-doped random fiber laser (YRFL) can be realized [15], more details can be found in Ref.

[15]. Subsequently, the YRFL seed is injected into another 6 m-long ytterbium-doped fiber pumped by a 976 nm diode laser through a 1:1 coupler (II) to boost its optical power. The amplified YRFL then serves as Raman pump for cascaded random Raman fiber lasing generation in a 5 km-long dispersion shifter fiber. Another 1:1 coupler (III)-based fiber looper mirror is used to provide broadband point feedback for cascaded random Raman fiber lasing. By adjusting the amplified YRFL laser wavelength and power, tunable 4 th and 5 th RRFL can be generated with a spectral tuning range of 1280-1360 nm and 1360-1450 nm, respectively [16].

In the DFG stage, the combined tunable RRFL and EDFA sources with WDM (reflection port: 1550-1700 nm, pass port: 1450-1490 nm) are first collimated with a broadband fiber collimator (FC). A mechanical chopper is placed at the beam path with a chopping frequency of 1 kHz. Subsequently, the laser beam is focused by a $CaF_2$ lens with a focal length of 100 mm into the ZGP waveguide. The beam diameters at focus are estimated as ~ 29 μm and 33 μm for the typical pump wavelength at 1360 nm and signal wavelength at 1565 nm, respectively. Since the RRFL and EDFA sources are both nonpolarized, the specific linearly polarized components of each beam are effectively involved in DFG. The combined pump/signal source features before ZGP waveguide are characterized by a spectral analyzer (Yokogawa AQ6370D) and a power meter (Ophir, 3A). The generated c. w. mid-infrared light after ZGP waveguide is first collimated by an uncoated ZnSe lens with a 25 mm focal length and then passes through an AR-coated Germanium filter which is used to block the residual pump/signal wave. For the spectral measurement, the mid-infrared light is focused by a ZnSe lens (3-12 μm AR-coated) with a 50-mm focal length into a hollow-core MIR fiber (OptoKnowledge HF500MW), and then detected by a grating-scanning monochromator (Zolix Omni-λ500i) with a liquid nitrogen cooled HgCdTe detector (Judson, DMCT16-De01).

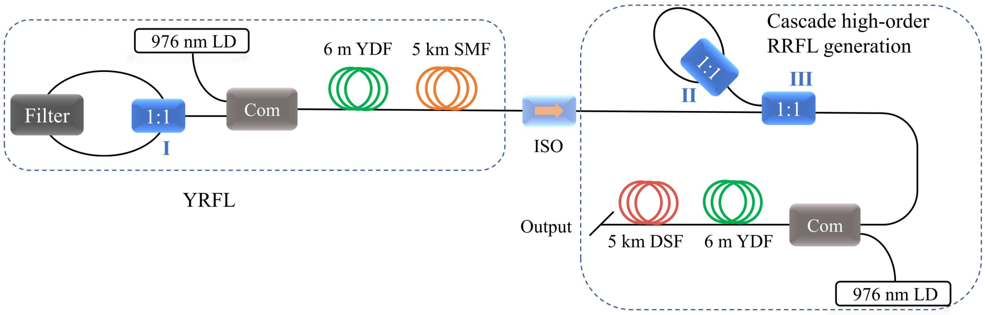


**Supplementary Figure 5** The setup of home-built tunable cw RRFL across the wavelength of 1280-1380 nm. I, II and III represent 1:1 coupler. LD, laser diode; YDF, ytterbium-doped fiber; SMF, single mode fiber; DSF, dispersion shift fiber; Com, combiner; ISO, isolator.

## V. Supplementary Note 5: Detail characterization of the $\chi^{(2)}$ ZGP waveguide

In this section, detail characterizations of the $\chi^{(2)}$ ZGP waveguide, especially the transmission loss and surface roughness are conducted. Cut-back measurement technique [17] is used for waveguide transmission loss calibration. Two waveguide samples with different length of 8 mm and 10 mm are employed. The input pump wave (2.4 μm) power is set as 60 mW. The averaged insertion losses of the 10 mm and 8 mm waveguides are measured as 10.24 dB and 10.08 dB, respectively, obtained by five repeated measurements. Thus, the transmission loss of the $\chi^{(2)}$ ZGP waveguide at the 2.4 μm pump wavelength in TM polarization is obtained as 0.8 dB/cm. Similar measurement process is executed for the signal wave at a wavelength of 3.4 μm in TM polarization, which shows a transmission loss of 1.2 dB/cm.

In addition, a 3-dimensional characterization of the ZGP waveguide by using the laser microscope (Olympus, OSL5000) is conducted. The measurement zone area is ~ 643 × 644 μm$^2$ with a 20× objective lens. As shown in Supplementary Figure 6(a, c), the width and depth of the groove is measured as 30.5 μm and 54.4 μm, respectively. To accurately characterize the microscopic profile of the groove sidewall, the ZGP waveguide is tilted by 30 degrees (Supplementary Figure 7(b, d)). In the orientation parallel to the micro-grooves, the line roughness of the side wall is measured as 0.596 μm. Meanwhile, an average line roughness of 0.595 μm is obtained by measuring eight grooves, and the standard deviation is calculated as 0.051 μm. We therefore suggest that with a surface roughness of $< 0.6$ μm, the waveguide scattering loss could be small in the LWIR region, and no significant influence is imposed on the waveguide mode and PM condition.

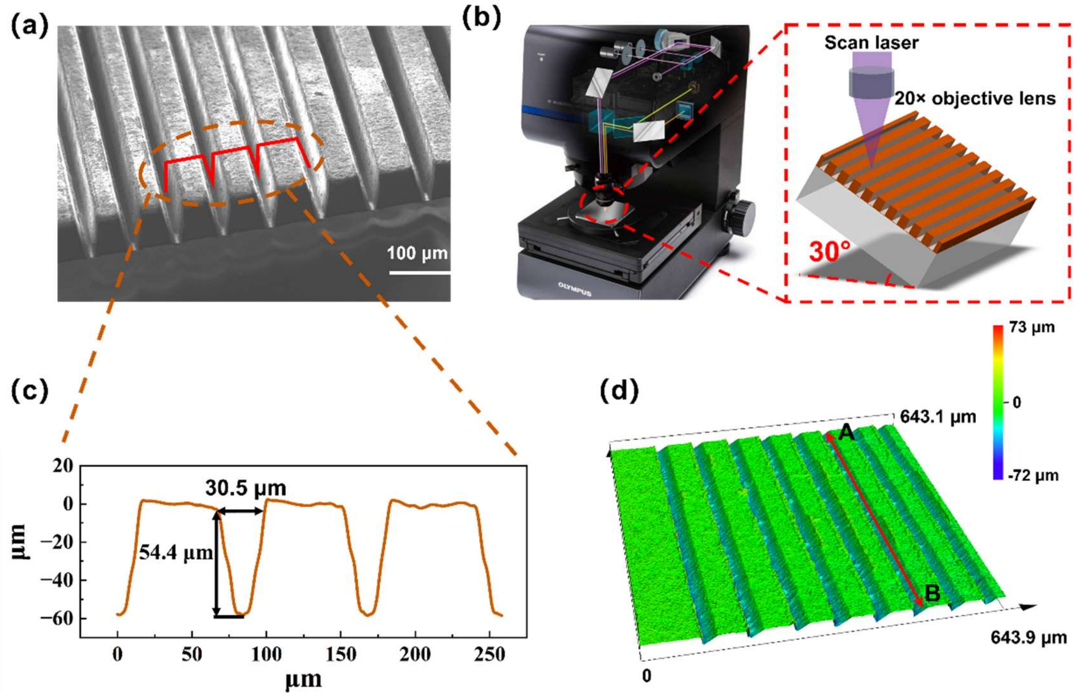


**Supplementary Figure 6.** The 3-dimensional characterization of the waveguide surface roughness. (a) The scanning-electron image of the $\chi^{(2)}$ ZGP waveguide. (b) The measurement apparatus: laser microscope (Olympus, OSL5000), and the schematic of the microscopic profile measurement of the groove sidewall by tilting the ZGP waveguide by 30 degrees. (c) The measured cross-sectional profile of the $\chi^{(2)}$ ZGP waveguide. (d) The measured three-dimensional surface profile of the $\chi^{(2)}$ ZGP waveguide. An average line roughness between points A and B on the micro-groove side wall by measuring eight grooves, revealing an averaged roughness value of 0.596 μm. The baseline of 0 μm is set based on the original surface of the ZGP crystal. The sampling length is 639.4 μm.

## VI. Supplementary Note 6: Comparison of MIR OPG from the waveguide structures based on PPLN, OP-GaAs, and ZGP material platforms.

Here, a comparison of MIR OPG from waveguide structures based on PPLN, OP-GaAs and ZGP material platforms are presented in Supplementary Table 2. The OPG in the PPLN waveguide reported in Ref. 12 exhibits extraordinary threshold energy, but a spectral range of 1.7 to 2.7 μm, limited by the transparency window of the PPLN crystal. For the OPG in the OP-GaAs waveguide (Ref. 15), the idler wavelength spans from 9 to 12 μm, however, the threshold energy and quantum conversion efficiency are not reported. For the ZGP waveguide facilitated with the birefringence PM, reported in this work, LWIR spectrum spanning from 5 to 11 μm is realized, and a remarkable quantum efficiency of 74% is demonstrated operating at LWIR region.

**Supplementary Table 2.** The comparison of PPLN waveguide, OP-GaAs waveguide, and the demonstrated $\chi^{(2)}$ birefringence waveguide

| Crystal platform | Phase-matching mechanism | OPG wavelength (μm) | Length (mm) | Waveguide width (μm) | Threshold energy (pJ) | Quantum conversion efficiency (%) | Reference |
|---|---|---|---|---|---|---|---|
| PPLN | Quasi-phase matching | 1.7-2.7 [a] | 6 | 1.85 | 0.06 | 22.2% | 12 |
| OP-GaAs | Quasi-phase matching | 9-12 [b] | 14.5 | 10 | NA | N.A. | 15 |
| ZGP | Birefringence phase matching | 7-9[a]<br>5-11[c] | 10 | 40 | 616 | 74% | This work |

[a] idler wavelength range at -10 dB level, [b] idler wavelength range at full width, and [c] idler wavelength range at -30 dB level.

## VII. Supplementary Note 7: Input coupling efficiency characterization.

In the manuscript, the input coupling efficiency is evaluated by using the pinhole measurement. Here, the accuracy of pinhole measurement is checked by adopting the insertion loss technique to evaluate the coupling loss. At the pump wavelength of 2.4 μm with the TM polarization, the insertion loss which is consisted of the coupling loss, Fresnel loss, and waveguide scattering loss is measured. When an input power of 60 mW is used, an output power of 5.67 mW is obtained with an insertion loss measured as 10.24 dB. The Fresnel reflection loss is calculated to be 1.36 dB. In addition, cut-back measurement technique [17] is used for waveguide scattering loss calibration. Two waveguide samples with different length of 8 mm and 10 mm are employed. The input pump wave (2.4 μm) power is set as 60 mW. The averaged losses of the 10 mm and 8 mm waveguides are measured as 10.24 dB and 10.08 dB, respectively, obtained by five repeated measurements. Thus, the propagation loss including scattering loss of the $\chi^{(2)}$ ZGP waveguide at the 2.4 μm pump wavelength in TM polarization is obtained as 0.8 dB/cm. Thus, the coupling loss is calculated to be 10.24 - 1.36 - 0.8 = 8.08 dB. Therefore, we could conclude that the coupling efficiency is 15.6%, which is similar to the value (15.3%) obtained by the pinhole measurement method. It is worth mentioning that as in the insertion loss measurement, both the fundamental and higher-order modes output are collected, the mode-mismatch loss is not counted, and the coupling efficiency includes light modes coupled to both the fundamental and higher-order modes in the $\chi^{(2)}$ ZGP waveguide.

In addition, to evaluate the accuracy of pinhole measurement, repeated measurements for 6 times are conducted with the same input power of the 2.4 μm pump wave. As shown in Supplementary Figure 7, the measured minimum and maximum coupling efficiencies are 14.2% and 16.7%, respectively, corresponding to quantum conversion efficiencies in the range of 67.8% to 79%. An average quantum conversion efficiency is obtained as 73%, which is close to our claimed number. Hence, considering the above all, we believe that claimed quantum conversion efficiency is reliable.

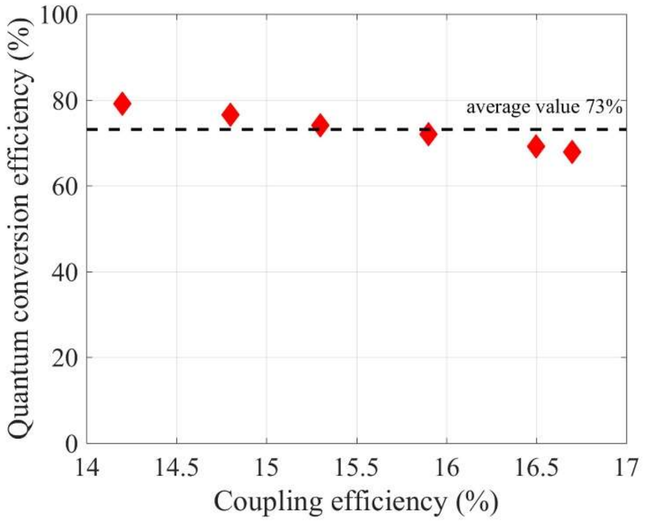


**Supplementary Figure 7.** The repeated coupling efficiency measurement for six times by using the pinhole method, and the corresponding quantum conversion efficiencies.

## Supplementary References


[1] Sanchez, D. et al. 7 μm, ultrafast, sub-millijoule-level mid-infrared optical parametric chirped pulse amplifier pumped at 2 μm. Optica 3, 147-150 (2017).

[2] Migal, E. A. et al. Highly efficient optical parametric amplifier tunable from near-to mid-IR for driving extreme nonlinear optics in solids. Opt. Lett. 42, 5218-5221 (2017).

[3] Liu, K. et al. Multimicrojoule GaSe-based midinfrared optical parametric amplifier with an ultrabroad idler spectrum covering 4.2-16 μm. Opt. Lett. 44, 1003-1006 (2019).

[4] Lesko, D. M. B. et al. A six-octave optical frequency comb from a scalable few-cycle erbium fibre laser. Nat. Photonics 15, 281-286 (2021).

[5] Liu, X. et al. Dry-etching-assisted femtosecond laser machining. Laser Photonics Rev. 11, 1600115 (2017).

[6] Nikogosyan, D.N. Nonlinear optical crystals: a complete survey, 1st ed. New York, NY: Springer, 2005.

[7] Li, W. et al. Theoretical Study on the Intrinsic Source of the Large Thermal Conductivity of Li-Based Chalcogenide Nonlinear Optical Crystals: From $AgGaS_2$ to $LiGaS_2$.

[8] Louisell, W. H. & Yariv, A. Quantum Fluctuations and Noise in Parametric Processes. I. Phys. Rev. 124, 1646-1654 (1961).

[9] Jankowski, M. et al. Dispersion-engineered $\chi^{(2)}$ nanophotonics: a flexible tool for nonclassical light. J. Phys. Photonics 3, 042005 (2021).

[10] Ledezma, L. et al. Intense optical parametric amplification in dispersion-engineered nanophotonic lithium niobate waveguides. Optica 9, 303-308 (2022).

[11] Hong, K. et al. Electric-field reconstruction of femtosecond laser pulses from interferometric autocorrelation using an evolutionary algorithm. Opt. Commun. 271, 169-177 (2007).

[12] Nam, S. et al. Octave-spanning mid-infrared femtosecond OPA in a $ZnGeP_2$ pumped by a 2.4 μm Cr: ZnSe chirped-pulse amplifier. Opt. Express 28, 32403-32414 (2020).

[13] Manzoni, C. et al. Optical-parametric-generation process driven by femtosecond pulses: Timing and carrier-envelope phase properties. Phys. Rev. A 79, 033818 (2009).

[14] Hinkelmann, M. et al. High-repetition rate, mid-infrared, picosecond pulse generation with μJ-energies based on OPG/OPA schemes in 2-μm-pumped $ZnGeP_2$. Opt. Express 28, 21499-21508 (2020).

[15] Hu, H. Wu, H. Tian, K. and Liang, H. Continuous-wave 2.9–3.8 μm random lasing via temperature-tunning free difference-frequency generation of random fiber lasers in PPLN crystal. Sci. China Inf. Sci. 66, 189401:2 (2023).

[16] Zhang, L. Jiang, H. Yang, X. Pan, W. and Feng, Y. Ultra-wide wavelength tuning of a cascaded Raman random fiber laser. Opt. Lett. 41, 215 (2016).

[17] Hunsperger, R. G. Integrated Optics: Theory and Technology (New York: Springer, 2009).
